# Corrigenda

**DOI:** 10.1080/16549716.2017.1377402

**Published:** 2017-09-08

Mutebi A, Muhumuza Kananura R, Ekirapa-Kiracho E, Bua J, Namusoke Kiwanuka S, Nammazi G, Paina L, Tetui M. Characteristics of community savings groups in rural Eastern Uganda: opportunities for improving access to maternal health service. Global Health Action. 2017;10(S4):1347363.

10.1080/16549716.2017.1347363

In the results section, the reference to table 1 has been corrected to table 2, the reference to table 2 corrected to table 3, and the reference to table 3 corrected to table 2. Also in the results section, 93% has been corrected to 94% and table 2 has been referenced on two further occasions. The author name ‘Getrude Nammazi’ has also been corrected to ‘Gertrude Nammazi’.

Muhumuza Kananura R, Tetui M, Bua J, Ekirapa-Kiracho E, Mutebi A, Namazzi G, Namusoke Kiwanuka & Peter Waiswa. Effect of a participatory multisectoral maternal and newborn intervention on birth preparedness and knowledge of maternal and newborn danger signs among women in Eastern Uganda: a quasi-experiment study. Global Health Action. 2017;10(S4):1362826.

10.1080/16549716.2017.1362826

In the results section of the abstract, (DID = 4.7) has been corrected to (DID = 5) to be consistent with the results in table 2. The text “whose p-value was <2.5% were considered for multivariate analysis” has been corrected to “whose p-value was less than or equal to 25% were considered for multivariate analysis”.

Ekirapa-Kiracho E, Muhumuza Kananura R, Tetui M, Namazzi G, Mutebi A, George A, Paina L, Waiswa P, Bumba A, Mulekwa G, Nakiganda-Busiku D, Lyagoba M, Naiga H, Putan M, Kulwenza A, Ajeani J, Kakaire-Kirunda A, Makumbi F, Atuyambe L, Okui A, Namusoke Kiwanuka S. Effect of a participatory multisectoral maternal and newborn intervention on maternal health service utilization and newborn care practices: a quasi-experimental study in three rural Ugandan districts. Global Health Action. 2017;10(S4):1363506.

10.1080/16549716.2017.1363506

The note to table 4 “Omitted under skilled delivery model (Model 3) because its p value was less than 2.5% in univariate analysis” has been corrected to “Omitted under skilled delivery model (Model 3) because its p value was greater than 25% in univariate analysis”.

Ajeani J, Ayiasi RM, Tetui M et al. A cascade model of mentorship for frontline health workers in rural health facilities in Eastern Uganda: processes, achievements and lessons. Global Health Action. 2017;10(S4): 1345497.

10.1080/16549716.2017.1345497

When the above article was first published online, Rornald Muhumuza Kananura’s name was spelt incorrectly as ‘Ronald Muhumuza Kananura’. This error has now been corrected.

